# Problematic internet use among young and adult population in Bangladesh: Correlates with lifestyle and online activities during the COVID-19 pandemic

**DOI:** 10.1016/j.abrep.2020.100311

**Published:** 2020-11-05

**Authors:** Md. Saiful Islam, Md. Safaet Hossain Sujan, Rafia Tasnim, Most. Zannatul Ferdous, Jakir Hossain Bhuiyan Masud, Sourav Kundu, Abu Syed Md. Mosaddek, M. Shahabuddin K. Choudhuri, Kagan Kircaburun, Mark D. Griffiths

**Affiliations:** a Department of Public Health and Informatics, Jahangirnagar University, Savar, Dhaka 1342, Bangladesh; b Youth Research Association, Savar, Dhaka 1342, Bangladesh; c Quest Bangladesh, Lalmatia, Dhaka 1207, Bangladesh; d Public Health Informatics Foundation (PHIF), Mirpur, Dhaka 1216, Bangladesh; e Advanced Institute of Industrial Technology, Shinagawa City, Tokyo 140-0011, Japan; f Department of Pharmacology, Uttara Adhunik Medical College, Uttara, Dhaka 1230, Bangladesh; g Department of Pharmacy, Jahangirnagar University, Savar, Dhaka 1342, Bangladesh; h United States Pharmacopeial Convention (USP) Herbal Medicines Compendium South Asia Expert Panel Member, Hyderabad, India; i International Gaming Research Unit, Psychology Department, Nottingham Trent University, 50 Shakespeare Street, Nottingham NG1 4FQ, UK

**Keywords:** Problematic internet use, Gaming, Social media use, COVID-19, Pandemic, Bangladesh

## Abstract

**Background:**

Although internet use can boost individuals’ quality of life in various aspects, activities on the internet (e.g., gambling, video gaming, social media use, pornography use, etc.) can be used as coping strategy to deal with psychological stressors and mood states (e.g., fear, anxiety, depression) particularly during the global COVID-19 pandemic.

**Objectives:**

The present study assessed problematic internet use (PIU) among Bangladeshi youth and adults in Bangladesh and examined its correlation with lifestyle and online activities during the COVID-19 pandemic.

**Methods:**

An online cross-sectional survey was utilized between May and June 2020 comprising 13,525 Bangladeshi individuals (61.3% male; age range 18–50 years; mean age 23.7 years) recruited from various online platforms. The self-report survey included questions concerning socio-demographics, lifestyle, and online activities during the COVID-19 pandemic, as well as psychometric scales such as the nine-item Internet Disorder Scale-Short Form (IDS9-SF).

**Results:**

Utilizing hierarchical regression analysis, problematic internet use was significantly and positively associated with those who were younger, having a higher level of education, living with a nuclear family, engaging in less physical exercise, avoiding household chores, playing online videogames, social media use, and engaging in recreational online activities.

**Conclusions:**

Excessive internet use appears to have been commonplace during the COVID-19 pandemic period and young adults were most vulnerable to problematic internet use.

## Highlights

• Largescale data of problematic internet use (PIU) during the pandemic are lacking.
• PIU was examined among a sample of 13,525 Bangladeshi participants.
• PIU was associated with being younger and having a higher education level.
• PIU was also associated with cigarette smoking, more sleep, and less physical exercise.
• PIU was associated with specific online activities (gaming, social media use).

## 1 Introduction

The novel coronavirus disease-2019 (COVID-19) pandemic has impacted most of the world with (at the time of writing) over 14.5 million cases and over 655,000 fatalities in 216 countries, creating a public health emergency of international concern (World Health Organization, 2020, Worldometer, 2020). To date, in Bangladesh (where the present study was carried out), there has been over 232,000 cases and over 3000 deaths (Institute of Epidemiology Disease Control and Research, 2020, Worldometer, 2020) from the first case identified on 8 March 2020 (Ferdous et al., 2020, Tasnim et al., 2020). To reduce the spread of the virus the Bangladesh government imposed strict social isolation and home quarantine measures.

Given the measures that have been implemented, it is unsurprising that individuals have turned to technology to help cope and function in this situation. Evidence has shown that internet use can enable individuals to achieve a higher perceived quality of life by supporting their jobs, education, and communication (Tran et al., 2018). Some internet-based applications are appropriate and easy to use, providing e-health care facilities (for instance detailed information regarding doctors, hospitals, and ambulance services, as well as some primary treatments or guidance for minor diseases), and education to individuals with low levels of health literacy. It has also been found that internet use can enhance the perceived quality of life by providing positive connections on social media, online shopping, online conferencing, and ongoing contact with friends and family living far away or in other countries (Pontes, Szabo, & Griffiths, 2015).

According to the Bangladesh Telecommunication Regulatory Commission (BTRC), the total number of internet subscribers was over 99 million at the end of January 2020 (BTRC, 2020). However, measures to inhibit the spread of COVID-19 (e.g., spatial distancing, social isolation, and home quarantine), as well as social and economic consequences, can also have a negative psychological impact with feelings of sadness, anxiety, fear, anger, annoyance, frustration, guilt, hopelessness, boredom, and panic (Ahorsu et al., 2020, Banerjee, 2020, Islam et al., 2020, Islam et al., 2020). A recent study conducted among Bangladeshi university students found that the prevalence of moderate to extremely severe depression, anxiety, and stress were 62.9%, 63.6% and 58.6%, respectively (Islam, Sujan, et al., 2020). To help cope with negative mood states, individuals may engage in psychoactive substance use or engage in specific online behaviors such as gambling, video gaming, streaming films and television shows, social media use, watching pornography and these potentially addictive behaviors may help to ease the stress of daily living (often referred to as ‘escapism’) and avoid problems and difficult thoughts (Blasi et al., 2019).

Consequently, the tendency to engage in such behaviors as putative coping mechanisms in emergencies such as the COVID-19 pandemic is likely to have increased considerably and can develop into patterns that are difficult to stop (King, Delfabbro, Billieux, & Potenza, 2020). For the majority of individuals, involvement in the aforementioned specific online activities provides positive benefits. However, for a small minority, excessive use of these activities can lead to severe problems and increase the risk of disordered or addictive use (Griffiths and Szabo, 2014, Pontes et al., 2015, Vismara et al., 2020). Despite the many inherent advantages afforded by internet use, excessive and uncontrolled use has become a significant social and behavioral problem. Problematic internet use (PIU) in its most extreme form has been termed ‘internet addiction’ defined as excessive and poorly controlled preoccupation, comprising urges and behaviors related to internet use that leads to clinical impairment and distress (Shaw & Black, 2008). PIU can lead to major distress and/or deterioration in financial, familial, social, educational and/or occupational domains (World Health Organization, 2019). It is therefore imperative that such behaviors, especially during the pandemic, remain at a moderate and regulated level. This is also significant, as gambling, gaming, and pornography industries may enable their clientele to invest longer periods in these activities via the use of opportunistic marketing campaigns (Király et al., 2020). The internet has become indispensable for most individuals, especially teenagers and young adults, but for a small minority, it can also become dysfunctional and cause difficulties, which can have negative consequences on the well-being of individuals (Machimbarrena et al., 2019). Research has indicated that the majority of youth aged between 18 and 29 years in many different countries have internet access (Joshi, Stubbe, Li, & Hilty, 2019).

The higher exposure by individuals to smartphones and the internet increases the chances of traditional offline psychosocial problems (bullying, verbal abuse, addictions, phobias) being experienced in their online equivalents (e.g., cyberbullying, sexting, internet addiction, nomophobia, etc.) (Machimbarrena et al., 2018). Choi and Lee (2015) noted that the abuse of smartphones can lead to many problems, including the dependence on social media, nomophobia, and PIU and that this can affect individual's psychological, social, academic and/or professional life. Nomophobic individuals constantly check the use of texts or calls and experience distress and difficulty if they are out of reach of their mobile phones (Bragazzi & Del Puente, 2014). Researchers have also claimed that individuals who spend a lot of time on the internet can suffer from nomophobia (Lachmann, Sariyska, Kannen, Stavrou, & Montag, 2017), which can decrease perceived quality of life (Foerster & Röösli, 2017). As a result of technology-related symptomology (e.g., nomophobia, cyberchondria, online addictions, etc.), individuals can experience long-term sleep problems, physical inactiveness, difficulties in focusing on work, and poorer relationships with family members (Casale, Lecchi, & Fioravanti, 2015).

PIU can be a risk factor for decreased quality of life, and the research in the area stretches back over 20 years (Guo et al., 2020) since the initial work of Griffiths, 1996, Young, 1996. Adolescents and young adults are at particularly high risk of PIU (Wartberg & Lindenberg, 2020). In some research, males have been said to be more likely to experience PIU than females (Laconi, Tricard, & Chabrol, 2015), although large-scale studies have found that males appear to experience more problems with online gaming whereas females appear to experience more problems with social media use (Andreassen et al., 2016).

A meta-analysis comprising 164 studies from 31 countries (N = 89,281) reported that the global pooled prevalence of PIU was 6% (average age = 18.42 years; SD = 5.02; age range 12–41 years) (Cheng & Li, 2014). In addition, Cheng and Li (2014) also reported that the prevalence of PIU in Asia was 7.1% which was lower than Middle East (10.9%) and North America (8%). Other *meta* analyses concerning internet addiction have reported multiple factors that are associated with internet use including being male (Su, Han, Jin, Yan, & Potenza, 2019), psychosocial factors (e.g., hostility, depression and anxiety, etc.) (Fumero et al., 2018, Tokunaga, 2017), and sleep problems (Alimoradi et al., 2019).

During the past three decades, the number of internet users has increased markedly in Bangladesh because the Bangladeshi government wants it to be a technologically developed country, and is known as the ‘Digital Bangladesh’ movement (Bangladesh Telecommunication Regulatory Commission, 2020). With the widespread use of the internet, potentially negative behaviors have been recorded in the country, including problematic use of the internet and internet addiction (Mamun, Rafi, et al., 2019). A few recent studies that have conducted in Bangladesh focusing on specific populations and specific areas. The prevalence rates of PIU have been reported to be 24% (among 573 graduate students aged 20–30 years from Dhaka University; Islam & Hossin, 2016), 27.1% (among 454 participants aged 19–35 years from three administrative divisions of Bangladesh; Hassan, Alam, Wahab, & Hawlader, 2020) and, 24% (among 350 high school students aged 13–17 years residing in Dhaka; Chandrima et al., 2020). However, these studies have all had small sample sizes. A Bangladeshi study by Islam and Hossin (2016) reported that PIU was associated with being male, having lower socioeconomic status, smoking cigarettes, being physically inactive, and experiencing psychological distress. Most recently, Hassan et al. (2020) reported that PIU among Bangladeshi citizens was significantly associated with living arrangements (e.g., living in a hostel), spending more time daily on the internet, having a detached family relationship, being physically inactive, and smoking cigarettes. In addition, other correlates of PIU in Bangladesh have included lower academic achievements, lower education in both mother and father, mother working outside the home, and spending more than two hours daily on the internet (Chandrima et al., 2020).

To date, there is no study that has examined PIU among youth and adults in Bangladesh during the COVID-19 pandemic period. Therefore, the present study investigated problematic internet use among this cohort and examined its’ association with lifestyle and online activities during COVID-19 pandemic. Based on previous Bangladeshi literature, it was hypothesized that those with PIU were more likely to be male, younger, have a higher level of education, be single, and live with a nuclear family. It was also hypothesized that there would positive correlations between PIU and lifestyle (e.g., smoking cigarettes, more or less sleep than the norm) and online behaviors (e.g., excessive internet browsing, and other online activities such as gaming, social media use, etc.) and negative correlations with measures of positive health (e.g., good sleep, regular exercise).

## 2 Methods

### 2.1 Participants and procedure

This present research utilized a cross-sectional survey comprising 13,525 individuals (61.3% male; age range 18–50 years; mean age 23.7 years), conducted between May and June 2020. A self-report questionnaire was developed and administered online. After generating an online survey link, the survey was shared in various online platforms (e.g., *Facebook, WhatsApp,* online blogs, etc.) in an effort to recruit as big a sample size as possible. The target group was the young adult population residing in Bangladesh who could speak and understand Bangla (although the survey was also completed by some individuals aged over 25 years). The inclusion criterion of the participants was that individuals had to be aged ≥18 years. The exclusion criteria included individuals who were minors and those who did not complete the full survey.

### 2.2 Sampling procedure

The sample size was calculated using the following equation:

$n=\frac{z^{2}pq}{d^{2}};n=\frac{1.96^{2}\times 0.271\times \left(1-0.271\right)}{0.0271^{2}}=1033.4\approx 1033$

Here,

n = number of samples

z = 1.96 (95% confidence level)

p = prevalence estimate (0.271)

q = (1-p)

d = Precession of the prevalence estimate (10% of 0.271)

Given the most recent study, Hassan et al. (2020) reported a PIU prevalence rate of 27.1% focusing on young adults in Bangladesh, a sample size of 1033 participants was estimated. Given the present study comprised 13,525 participants recruited via online convenience sampling, the sample size was more than sufficient in the present study.

### 2.3 Dependent and independent variables

In the present study, items concerning socio-demographic factors, lifestyle behaviors, and internet-related activities engaged in during the COVID-19 pandemic were the independent variables. The score on the psychometric scale assessing PIU was the dependent variable. A pilot test was conducted on 70 individuals, to test the understanding of the questionnaire. The data from the pilot survey were not included in the final analysis.

#### 2.3.1 Socio-demographic measures

Socio-demographic information was collected by asking questions regarding gender (male/female), age (later categorized: young [18–25 years] versus older [25 + years]), educational qualification (intermediate or below, bachelor degree, and higher education [above bachelor degree]), marital status (unmarried, married, and divorced), family type (nuclear versus joint/extended), monthly family income (later categorized: lower-income [<15,000 Bangladeshi Taka (BDT)], middle-income [15,000–30,000 BDT], and high-income [>30,000 BDT]), and residence (urban versus rural).

#### 2.3.2 Lifestyle related measures

Information regarding lifestyle-related behaviors during the pandemic period was collected including current cigarette smoking status (yes/no), average number of daily sleeping hours (classified as normal [7–9 h], less than normal [<7h], or more than normal [>9h] based on [Hirshkowitz et al., 2015, Islam et al., 2020]), daily physical exercising (yes/no), and engaging household chores (yes/no).

#### 2.3.3 Internet-related measures

Internet-related information was collected including the average number of internet browsing hours daily (later categorized: <2 h, 2–4 h, 5–6 h, and > 6 h). In addition, ‘yes/no’ questions were asked regarding which online activities were engaged in during the home quarantine (e.g., attending online classes, playing online games, social media use, and recreational activities such as watching films and television series).

#### 2.3.4 Problematic internet use

The IDS9-SF is one of the most robust psychometric screening tools for assessing problematic internet use and was developed by Pontes and Griffiths (2016). It has been validated in over 15 different languages including Persian (Lin et al., 2018), Turkish (Bener, Griffiths, Baysoy, Catan, & Yurtseven, 2019), and Italian (Soraci et al., 2020). The present study used the Bangla version (Islam, Rahman, et al., 2020). The scale comprises nine questions regarding problematic internet use (e.g., *“Have you jeopardized or lost an important relationship, career or an educational opportunity because of your online usage?”*) which are responded to on a five-point Likert scale ranging from 1 (*Never*) to 5 (*Very often*). Total scores are obtained by summing the raw scores of each item and total scores range from 9 to 45, with higher scores indicating a higher degree of problematic internet use. In the present study, the Cronbach’s alpha of IDS9-SF indicated very good internal consistency (α = 0.85).

### 2.4 Data analyses

The data were coded and analyzed using two statistical software packages (Microsoft Excel 2019, and IBM SPSS Statistics version 25). Microsoft Excel was used to perform data cleaning, coding, editing, and sorting. An Excel file including all variables was imported in SPSS software. Descriptive statistics (e.g., frequencies, percentages, means, standard deviations, etc.) were performed using SPSS software. In addition, *t*-tests or one-way ANOVA tests were performed to determine significant relations of the mean IDS9-SF scores with all examined variables applying Bonferroni correction (by dividing the *p*-value significance threshold [0.05] by the number of statistical tests, the *p*-value for significance was calculated to be *p* < .003). Finally, demographic, lifestyle and internet-related variables that significantly differed in terms of PIU scores, were included into hierarchical regression analysis with PIU as the dependent variable.

### 2.5 Ethics

The present study was carried out in accordance with the ethical guidelines for human involving investigations (i.e., Helsinki Declaration, 1975). In addition, formal ethics approval was granted by the research team’s Ethical Review Committee. Participants were well informed about the procedure and purpose of the study, and the confidentiality of their information. All data were collected anonymously, and all participants provided informed consent.

## 3 Results

### 3.1 Characteristics of the sample

The participants’ general characteristics are summarized in Table 1. A sizeable majority of participants had a bachelor degree level of education (68.0%), were unmarried (83.1%), came from nuclear families (78.3%), were from upper-income families (44.7%), and resided in an urban area (61.9%). During the pandemic period, the majority did not smoke cigarettes (84.1%), two-thirds slept in a normal daily range of 7–9 h (68.0%), half engaged in regular exercise (51.1%), and a quarter did not engage in household chores (23.3%).

**Table 1:** Descriptive analysis of each variable and association with IDS9-SF scores.

**Variables**	**Total N = 13525**		**IDS9-SF scores (M = 22.7; SD = 7.8)**				
	**n**	**(%)**	**Mean**	**SD**	**95% CI for Mean**	**t/F**	***p*-value**
**Socio-demographic factors**							
**Gender**							
Male	8287	(61.3)	22.7	(7.7)	(22.5–22.9)	0.15	0.696
Female	5238	(38.7)	22.8	(7.9)	(22.5–23.0)		
**Age**							
Young (18–25)	10,656	(78.8)	23.1	(7.8)	(22.9–23.2)	112.81	<0.001
Adult (>25 years)	2869	(21.2)	21.4	(7.3)	(21.1–21.6)		

**Educational qualification**							
Intermediate (11–12) or below	2474	(18.3)	22.2	(7.9)	(21.9–22.5)	21.56	<0.001
Bachelor	9196	(68.0)	23.0	(7.8)	(22.9–23.2)		
Higher education (above bachelor)	1855	(13.7)	21.9	(7.2)	(21.6–22.3)		

**Marital status**							
Unmarried	11,243	(83.1)	23.1	(7.8)	(22.9–23.2)	67.91	<0.001
Married	2219	(16.4)	21.0	(7.4)	(20.7–21.3)		
Divorced	63	(0.5)	21.7	(8.3)	(19.6–23.8)		

**Family type**							
Nuclear	10,588	(78.3)	22.9	(7.8)	(22.7–23.0)	24.07	<0.001
Join	2937	(21.7)	22.1	(7.7)	(21.8–22.4)		

**Monthly family income**							
Lower class	2612	(19.3)	22.4	(7.9)	(22.1–22.7)	5.22	0.005
Middle class	4869	(36.0)	23.0	(7.8)	(22.8–23.2)		
Upper class	6044	(44.7)	22.6	(7.7)	(22.5–22.8)		

**Residence**							
Urban area	8376	(61.9)	22.8	(7.7)	(22.7–23.0)	5.61	0.018
Rural area	5149	(38.1)	22.5	(7.8)	(22.3–22.7)		

**Lifestyle factors**							
**Smoking status**							
Yes	2152	(15.9)	23.3	(7.5)	(23.0–23.6)	12.77	<0.001
No	11,373	(84.1)	22.6	(7.8)	(22.5–22.8)		

**Sleeping hours**							
Less than normal	2638	(19.5)	23.2	(7.5)	(22.9–23.4)	108.61	<0.001
Normal (7–9 h)	9191	(68.0)	22.2	(7.7)	(22.0–22.3)		
More than normal	1696	(12.5)	25.1	(7.9)	(24.7–25.5)		

**Physical exercise**							
Yes	6915	(51.1)	21.8	(7.4)	(21.7–22.0)	184.11	<0.001
No	6610	(48.9)	23.6	(8.0)	(23.5–23.8)		

**Doing household chores**							
Yes	10,378	(76.7)	22.3	(7.6)	(22.1–22.4)	154.11	<0.001
No	3147	(23.3)	24.2	(7.9)	(23.9–24.5)		

**Online behaviors**							
**Internet browsing hours**							
<2 h	1624	(12.0)	18.6	(7.2)	(18.3–19.0)	476.80	<0.001
2–4 h	4266	(31.5)	25.6	(7.8)	(25.4–25.9)		
5–6 h	4211	(31.1)	20.9	(7.2)	(20.7–21.1)		
>6 h	3424	(25.3)	23.3	(7.2)	(23.1–23.5)		

**Attending online class**							
Yes	4836	(35.8)	22.7	(7.7)	(22.4–22.9)	0.51	0.476
No	8689	(64.2)	22.8	(7.8)	(22.6–22.9)		

**Playing online games**							
Yes	4635	(34.3)	23.7	(7.7)	(23.5–23.9)	115.44	<0.001
No	8890	(65.7)	22.2	(7.7)	(22.0–22.4)		

**Social media purposes**							
Yes	8771	(64.9)	23.5	(7.7)	(23.4–23.7)	285.41	<0.001
No	4754	(35.1)	21.2	(7.7)	(21.0–21.4)		

**Recreational activities**							
Yes	10,547	(78.0)	23.0	(7.7)	(22.9–23.2)	66.22	<0.001
No	2978	(22.0)	21.7	(7.7)	(21.4–22.0)		

### 3.2 Mean scores of IDS9-SF and online behaviors

Analysis on the total scores of IDS9-SF demonstrated that the mean score was 22.7 out of 45 (SD = 7.8) with no significant gender differences. A quarter of the sample spent more than six hours daily on the internet (25.3%). Participants engaged in the following activities on the internet: attending online class (35.8%), using social media (64.9%), playing online videogames (34.3%), and recreational activities such as watching movies and television series (78.0%).

### 3.3 Association of variables with internet use disorder

With regard to socio-demographics, the mean scores of IDS9-SF were significantly higher among (i) young adults vs. older adults (23.1 ± 7.8 vs. 21.4 ± 7.3, *p* < .001), (ii) participants having bachelor degree vs. higher levels of education (23.0 ± 7.8 vs. 21.9 ± 7.2, *p* < .001), (iii) unmarried vs. married participants (23.1 ± 7.8 vs. 21.0 ± 7.4, *p* < .001), and (iv) participants from a nuclear family vs. joint families (22.9 ± 7.8 vs. 22.1 ± 7.7, *p* < .001) (see Table 1).

In addition, the mean scores of IDS9-SF were significantly associated with specific lifestyle activities during home quarantine. More specifically, IDS9-SF scores were significantly higher among (i) participants who currently smoked cigarette vs. those who did not (23.3 ± 7.5 vs. 22.6 ± 7.8, *p* < .001), (ii) participants who slept more than nine hours daily vs. those who slept in the normal range (7–9 h) (25.1 ± 7.9 vs. 22.2 ± 7.7, *p* < .001), (iii) participants who did not engage in physical exercise vs. those who did (23.6 ± 8.0 vs. 21.8 ± 7.4, *p* < .001), and (iv) participants who did not engage in household chores vs. those who did (24.2 ± 7.9 vs. 22.3 ± 7.6, *p* < .001).

Furthermore, the mean scores of IDS9-SF were significantly associated with online activities during the home quarantine, and significantly higher among (i) participants who browsed more on the internet daily (2–4 h) vs. those who browsed the internet<2 h daily (25.6 ± 7.8 vs. 18.6 ± 7.2, *p* < .001), (ii) participants who played online video games vs. those who did not (23.7 ± 7.7 vs. 22.2 ± 7.7, *p* < .001), (iii) participants who used social media vs. those who did not (23.5 ± 7.7 vs. 21.2 ± 7.7, *p* < .001), and (iv) participants who engaged in recreational activities on the internet vs. those who did not (23.0 ± 7.7 vs. 21.7 ± 7., *p* < .001). In the present sample, after implementing a Bonferroni correction (*p* < .003), IDS9-SF scores did not significantly differ according to monthly income (23.0 ± 7.8 vs. 22.4 ± 7.9, *p* = .005) and residence (22.8 ± 7.7 vs. 22.5 ± 7.8, *p* = .018).

### 3.4 Hierarchical regression

The results of hierarchical regression analysis are presented in Table 2. Factors that were statistically significant in the group difference analyses (*t*-tests and ANOVA) were included in a hierarchical regression analysis. Socio-demographic factors (i.e., age, educational qualification, marital status, and family type) were included in Block 1. Lifestyle factors (i.e., smoking status, sleeping hours, physical exercise, and doing household chores) comprised Block 2. In Block 3, online behaviors (i.e., internet using hours, playing online games, social media purposes, and recreational activities) were included. After implementing a Bonferroni correction, problematic internet use was positively associated with lower age (β = −0.07, *p* < .001), having higher education (β = 0.03, *p* < .001), living with nuclear family (β = -0.03, *p* < .003), lower physical exercise (β = 0.11, *p* < .001), avoiding household chores (β = 0.09, *p* < .001), playing online games (β = −0.05, *p* < .001), social media use (β = −0.12, *p* < .001), and engaging in recreational online activities (β = −0.04, *p* < .001). Consequently, marital status, smoking status, sleeping hours, and number of hours of daily internet use were not significant in the hierarchical regression analysis. It should also be noted that the predictive effects of education level, family type, playing online videogames, and recreational online activities were very small. The regression model predicted 6% of the variance in problematic internet use (*F*_12,13512_ = 72.42, *p* < .001).

**Table 2:** Hierarchical regression analysis predicting problematic internet use.

Model	B	SE	β	*t*	ΔR^2^
**Block 1 - Socio-demographics** (*F*_(4,13520)=_52.88; *p* < .001)					0.02
Age	−0.12	0.02	−0.07	−6.77**	
Educational qualification^a^	0.43	0.12	0.03	3.50**	
Marital status^b^	−0.42	0.21	−0.02	−2.07	
Family type^c^	−0.47	0.16	−0.03	−2.93*	

**Block 2 - Lifestyle factors** (*F*_(4,13516)=_77.14; *p* < .001)					0.02
Smoking status^d^	−0.51	0.18	−0.02	−2.85	
Sleeping hours^e^	0.32	0.12	0.02	2.78	
Physical exercise^d^	1.68	0.13	0.11	12.55**	
Doing household chores^d^	1.70	0.16	0.09	10.71**	

**Block 3 – Online behaviors** (*F*_(4,13512)=_82.87; *p* < .001)					0.02
Internet using hours^f^	0.09	0.07	0.01	1.32	
Playing online games^d^	−0.86	0.14	−0.05	−6.07**	
Social media purposes^d^	−1.89	0.15	−0.12	−13.00**	
Recreational activities^d^	−0.83	0.17	−0.04	−5.01**	

Note: B = unstandardized regression coefficient; SE = Standard error; β = standardized regression coefficient; ^a^1 = Intermediate (11–12) or below, 2 = Bachelor, 3 = Higher education (above bachelor); ^b^1 = Unmarried, 2 = Married, 3 = Divorced; ^c^1 = Nuclear, 2 = Joint; ^d^1 = Yes, 2 = No; ^e^1=<7 h, 2 = 7 – 9 h, 3=>9 h; ^f^ 1=< 2 h, 2 = 2–4 h, 3 = 5–6 h, 4=> 6 h; *F*_(12,13512)_ = 72.42, *p* < .001, *R*^2^_Adj_ = 0.06; *p < .003, **p < .001.

## 4 Discussion

To the best of the authors’ knowledge, the present study is the first large-scale study among adults in Bangladesh assessing various factors associated with problematic internet use during the COVID-19 pandemic. PIU was significantly associated with being younger in age, having a bachelor degree level of education, being unmarried, being a member of a nuclear family, having middle-income socioeconomic status, living in an urban area, being a cigarette smoker, being a heavier sleeper, being physically inactive, not engaging in household chores, and having higher engagement with online activities (e.g., playing video games, social media use, and online recreational activities).

In modern society, the internet has become essential in people’s everyday lives but prolonged daily use can have a detrimental effect on individual’s physical and mental well-being. Since the mid-1990s, problematic internet use (PIU) has been thoroughly investigated including Asian countries (Islam & Hossin, 2016). As soon as the COVID-19 outbreak began in Bangladesh and the countrywide lockdown was declared by the government, many individuals had more leisure time to spend at home. This excessive leisure time is likely to have triggered boredom among many internet users and its excessive use during the pandemic may have facilitated problematic internet use behavior (Wang, 2019). There is significant evidence indicating that the health of a minority of adolescents and adults is adversely affected by PIU (Kuss, Griffiths, Karila, & Billieux, 2014).

### 4.1 Comparison with other studies

The findings of this study are now compared with the previous studies that have focused on problematic internet use or internet addiction in Bangladesh as well as globally (from meta-analysis). According to the present study, no gender differences were found which is similar with the previous findings in Bangladesh (Islam & Hossin, 2016). Previous studies in Bangladesh have reported that PIU is higher among males found than females (Mamun et al., 2019, Uddin et al., 2016)*,* although research among adolescents in Bangladesh reported that females were likely to report PIU compared to males (Afrin & Hossain, 2017). Furthermore, the present study’s findings are different to recent meta-analysis which found males experienced higher levels of PIU than females (Su et al., 2019).

The present study found that the level of PIU was higher among individuals from a nuclear family than individuals from an extended family, although those from a nuclear family are more likely to be lonely than those living in the joint/extended family (Antognoli-Toland, 2001), and according to a study in Bangladesh (Mamun et al., 2020) and previous meta-analysis, loneliness is associated with PIU and internet addiction (Tokunaga, 2017). In addition, the present study found that unmarried (single) individuals were more likely to experience PIU compared to those who were married. That may be because individuals who are separated from their families and/or not with a current partner may be using the internet to address their need for interpersonal interaction and to build substitute social networks and social support from virtual friendship which increases their use of internet.

Findings also indicated that PIU was higher among cigarette smokers than non-smokers, and that cigarette smoking was significantly associated with PIU. This finding was similar to previous studies in Bangladesh among graduate students (Islam & Hossin, 2016) and young adults (Mamun, Rafi, et al., 2019). A previous study examining internet addiction among adolescents reported that cigarette smoking significantly increased stress and social anxiety which can play a contributory role internet addiction (Feng, Ma, & Zhong, 2019).

Young individuals (aged 18–25 years) and those with a Bachelor’s degree had higher levels of PIU than older individuals and other educational levels. Most studies have found that internet use tends to be more problematic for younger users. Such individuals are likely to be using the internet for both educational and/or occupational purposes as well as for leisure pursuits. There may be several reasons why students are susceptible to PIU including (i) easy access to entertainment and social interaction features that are most enjoyed by this cohort (Ceyhan, 2011), (ii) environmental factors (e.g., freedom from parental control due to moving away from the family home for the first time) and psychological factors (loneliness, depression and anxiety) in the lives of students that may result in increased internet use (Bahrainian, Alizadeh, Raeisoon, Gorji, & Khazaee, 2014), (iii) students facing new problems adapting to university life and trying to make new friends and/or maintain old ones through the use of various online applications (Sharma, Sahu, Kasar, & Sharma, 2014), and (iv) using the internet as a form of stress relief (e.g., from the pressures of having to pass coursework and examinations. Moreover, the survey was carried out during the lockdown, a lot of educational and occupational work has moved online. Together these factors might help explain the relatively high levels of PIU in the present study.

Participants not engaging in regular physical exercise were more likely to experience PIU and supports findings from previous Bangladeshi studies (Hassan et al., 2020, Islam and Hossin, 2016). Research more generally has indicated that those experiencing PIU tend to have more sedentary lifestyles (Aşut et al., 2019, Griffiths, 2010). According to a recent study, healthy individuals experience less PIU than individuals who are obese. Such individuals are more likely to have sedentary lifestyles and are less likely than healthy individuals to engage in physical exercise (Bozkurt, Özer, Şahin, & Sönmezgöz, 2018). The present study also found that PIU was higher among those not engaging in household chores. Not engaging in household chores during countrywide lockdown and home quarantine is likely to have contributed to a more sedentary lifestyle and using the internet for activities such as social media use and online gaming which may have contributed to PIU.

More specifically, the regression analysis showed that those who used the internet for greater amounts of time was not associated with PIU (as has been found in prior Bangladeshi studies [e.g., Hassan et al., 2020, Mamun et al., 2019, Mamun et al., 2019] and elsewhere globally) but with specific types of online activity (i.e., online gaming, social media use) in the present study, and as found in a previous Bangladeshi study (Mamun et al., 2020). These activities are likely to have been even more prevalent during lockdown and quarantine (Király et al., 2020). The lack of regulation of internet use by individuals is significantly associated with PIU (Bruno et al., 2014). Online participation in these activities is often used as a tool to reduce individuals’’ social alienation, isolation, and loneliness.

The present study is in line with previous Bangladeshi studies showing that online communication, social media use, and using internet for recreation purposes are related to PIU (Mamun et al., 2020, Mamun et al., 2019). PIU appears to be a major health and psychosocial risk for a minority, and long online sessions (whether for educational and/or leisure purposes) increase physical inactivity and its consequences can include narrower lumbar intervertebral discs, increased fat content in the para-spinal muscles, and low back pain and disability (Teichtahl et al., 2015). Moreover, spending a lot of time engaged in online activities combined with buffering (e.g., the waiting time when downloading large amounts of data related to both work and leisure activities) has been reported as making some individuals more aggressive and hostile (Yen, Yen, Wu, Huang, & Ko, 2011). This can also have an impact on both physical and mental health.

The differences in the pattern of relationships between variables in the present study compared to other studies in Bangladesh and globally (from meta-analysis) may be due to different target populations, different screening instruments, lack of representative samples, and unprecedented factors relating lockdown situation due to COVID-19.

### 4.2 Limitations

There are some limitations in the present study that should be taken into account when interpreting the results. First, the analysis was cross-sectional in nature so did not determine the causality of any of the examined variables. A longitudinal study would be needed in this respect. Second, the study utilized an online self-report methodology which may be open to social desirability and memory recall biases. Although the sample was large-scale, a sizeable majority of students participated and the low average sample age demonstrates the sample was not nationally representative.

## 5 Conclusions

To the best of the present authors’ knowledge is, the present investigation is the first large-scale study in Bangladesh to investigate problematic internet use during the COVID-19 pandemic in Bangladesh. PIU was strongly associated with socio-demographic factors (being younger in age, having a bachelor degree level of education, being unmarried, being a member of a nuclear family, having middle-income socioeconomic status, living in an urban area), lifestyle factors (being a cigarette smoker, being a heavier sleeper, being physically inactive, not engaging in household chores), and online behaviors of an individual (internet browsing hours, playing online games, social media purposes, recreational activities).

In countries such as Bangladesh where the growth of internet use is more rapid than socio-economic development, the study of PIU is particularly important especially during the COVID-19 pandemic. The results from the study may assist mental health professionals in their clinical practice, particularly in the care of patients who are addicted to the internet. In addition, the findings of the present study may help policymakers to identify target groups of problematic internet users and help in the design of intervention programs to prevent excessive internet use. Furthermore, suitable preventive measures such as educating students, and the general public about safe internet use are needed, as well as counseling for those individuals already addicted to the internet.

## CRediT authorship contribution statement

**Md. Saiful Islam:** Conceptualization, Methodology, Investigation, Data curation, Formal analysis, Writing - original draft, Validation. **Md. Safaet Hossain Sujan:** Writing - original draft, Validation. **Rafia Tasnim:** Writing - original draft, Validation. **Most. Zannatul Ferdous:** Conceptualization, Supervision, Validation. **Jakir Hossain Bhuiyan Masud:** Validation. **Sourav Kundu:** Validation. **Abu Syed Md. Mosaddek:** Validation. **M. Shahabuddin K. Choudhuri:** Validation. **Kagan Kircaburun:** Validation, Formal analysis, Writing - original draft. **Mark D. Griffiths:** Writing - original draft, Editing, Supervision, Validation.

## Declaration of Competing Interest

The authors declare that they have no known competing financial interests or personal relationships that could have appeared to influence the work reported in this paper.
