# Supplementary material for: Problematic internet use among young and adult population in Bangladesh: Correlates with lifestyle and online activities during the COVID-19 pandemic
Source: Addict Behav Rep. 2020 Nov 5;12:100311. doi: 10.1016/j.abrep.2020.100311 (PMC7752719; doi:10.1016/j.abrep.2020.100311)
Supplement: Supplementary data 1 [file mmc1.docx]

| Name | Institution | Email |
| --- | --- | --- |
| Sk Kamruzzaman | Patuakhali Science & Technology University | sk.kamruzzaman1301@gmail.com |
| Alomgir Hossain | Rajshahi University | alamgir199817@gmail.com |
| Khairrun Nahar Pinky | University of Rajshahi | knpink99@gmail.com |
| Md Nazmus Sakib | Hamdard University Bangladesh | nazmusshakib0009@gmail.com |
| Tanziha Yeasmin Toma | Jahangirnagar University | Tanzihatoma1613@gmail.com |
| Abdul Ahad Nahin | Jahangirnagar University | ahadnahin2015@gmail.com |
| Md. Riad Islam | Rajshahi University | riadislam535@gmail.com |
| Md Abid Bin Siddique | Jahangirnagar University | abidbinsiddiq6@gmail.com |
| Ikram Hossen | Jahangir Nagar University | ikramhossenpranto@gmail.com |
| Fahim Shariar Anter | Noakhali Science & Technology University | fahimsontor99@gmail.com |
| Tanmoy Dutta | Jahangirnagar University | duttatanmoyju@gmail.com |
| Saleha Begum | Noakhali Science and Technology University | mashfikaaymon100@gmail.com |
| Farhan Sarwar | Jahangirnagar University | farhansarwar821@gmail.com |
| Rima Basak | Independent University | rimabasak381@gmail.com |
| Md Ebrahim Sheikh | Jagannath University | ebrahimmkt76@gmail.com |
| Tania Akter Neela | Noakhali science and technology university | tanianeela@gmail.com |
| Sanjida Ahmed | Jahangirnagar University | sanjidanisa802@gmail.com |
| Tazrin Ahmed Khan | University of Rajshahi | tazrinkhan97@gmail.com |
| Sristi Chowdhury | Noakhali Science and Technology University | sristichowdhury10@gmail.com |
| Tanjina Yousuf | Noakhali Science and Technology University | tanjinayousufjemi@gmail.com |
| Nazmus Sakib | Jahangirnagar University, Savar, Dhaka | nazmussakibju2011@gmail.com |
| Shaowni Das | Jahangirnagar University | Shaownidasneli@gmail.com |
| Emtius Hasnat Nishat | Shahidul Chowdhury Engineering College | emtiushasnat@gmail.com |
| Srabanti Anwar | Rajshahi University | shrabananwar@gmail.com |
| Reshma Afroz Rimi | Jahangirnagar University | reshmaafroz05@gmail.com |
| Fahmida Alam Chowdhury | Noakhali Science & Technology University | fahmidabristyswnstu@gmail.com |
| Ayesha Akter | University of Rajshahi | Ayesharahmanasha13@gmail.com |
| Sayma Islam Alin | Jahangirnagar University | sayma.alin@phiju.edu.bd |
| Shakib Al Hasan | Gono Bishwabidyalay | shakib.2858@gmail.com |
| Saminur Haque | University of Dhaka | saminurhaque332@gmail.com |
| Tanjina Akter Shamme | Jagannath university | angelshammi78@gmail.com |
| Imran Hosen | University of Rajshahi | imranruir14@gmail.com |
| Sajana Tahmid | North South University | sejanaq343@gmail.com |
| Samira Akter Siyam | Govt. College Of Home Economics | samiraaktersiyam655@gmail.com |
| Tanvir Sarker Tutul | Jahangirnagar University | tanvirsarkerju1998@gmail.com |
| Md. Saddam | Bangabandhu Sheikh Mujibur Rahman Science & Technology University | saddubmb@gmail.com |
| Sushmita Karmokar | Noakhali Science and Technology University | sushmitakarmokar907@gmail.com |
| Md Monirul Islam | Pharmasia Ltd | mislamgb@yahoo.com |
| Tasfia Bhuiyan | Noakhali Science and Technology University | tasfiaahmedt@gmail.com |
| Rifat Nowshin | Jahangirnagar University | rifatnowshin1612@gmail.com |
| Pinon Nath | Noakhali Science & Technology University | nathpinon96@gmail.com |
| Md Habibur Rahaman | Jagannath University | Habib.jnu1995@gmail.com |
| Mst. Jemi Hkatun | Jahangirnagar university | jemikhetlal98@gmail.com |
| Asmaul Husna Ritu | Jahangirnagar University | 268husna@gmail.com |
| Jahid Bin Sultan | Noakhali science and technology university | zahidsultan470@gmail.com |
| Sabiha Tasnim Reevny | Jahangirnagar University | STReevnyJU@gmail.com |
| Mridul Pathan | Independent University Bangladesh | mridulpathan17@gmail.com |
| Rezowan Ahmmed | Khulna University | 191448@ku.ac.bd |
| Rupa Akter | Independent University | [akterrupa919@gmail.com](mailto:akterrupa919@gmail.com) |
| Naimur Rahman | Jahangirnagar University | naimur634@gmail.com |
| Md Mujahidul Islam | Chittagong University | [mujahid19604032@gmail.com](mailto:mujahid19604032@gmail.com) |
| Most.Israt Jahan | Noakhali Science and Technology University | isratmunni2828@gmail.com |
| Afia Ayub | Tejgaon College, Dhaka | afia199720122016@gmail.com |
| Rubiya Afrin | Jahangirnagar University | afrinrubaiya9@gmail.com |
| A.K.M. Afzal Hossain | Bangladesh Agricultural University | a.k.m.afzalhossain92@gmail.com |
| Minhazul Islam Chowdhury | Southeast University | sworan007@gmail.com |
| Debashish Paul Deb | Independent university | debashispauldeb@gmail.com |
| Md Delwar Hossen | Independent University, Bangladesh | delwarhossen1097@yahoo.com |
| Sarup Das | Uttar Kattali Al-Haj Mostafa Hakim College | sarupdasnsn@gmail.com |
| Zakia Khanom Tisha | Jahangirnagar University | azakiatisha57@gmail.com |
| Mt. Shirajum Monira | Jagannath University | shirajumminora.jnu@gmail.com |
| Muhammad Rubel | Comilla University | ahmedrubel324@gmail.com |
| Shahadat Hossain Shakil | Noakhali Scienceand Technology | shshakil29@gmail.com |
| Shabrina Islam Mim | University of Barishal | sabrinamim.law5@gmail.com |
| Kifyat Tasnim | Jashore University of Science and Technology | kifyat110196@gmail.com |
| Fahmida Faiza | International Islamic University Chittagong | fahmidafaiza921@gmail.com |
| Md Naimul Islam Arif | University of Rajshahi | mr.arif3@yahoo.com |
| Sazzad Hossain | Noakhali Science and Technology University | sazzadhossainusama@gmail.com |
| Sajjad Bin Sogir | Jahangirnagar university | 321mdsajjad@gmail.com |
| Md.Shamsul Haque | Jagannath University | shamsulshanto69@gmail.com |
| Md Jabed Hossain | NSTU | mdjabedadnan@gmail.com |
| Md Belal Hossen | Rajshahi University | belal19599@gmail.com |
| Supti Podder | University of Dhaka | suptipodder1998@gmail.com |
| Md.Rezaul Karim | Noakhali Science &Technology University | mdrezaul1525@gmail.com |
| Arpita Chakrabarty | Jahangirnagar University | a.chakrabarty6116@gmail.com |
| Mahiare-Uz Zaman | Jahangirnagar University | mahiershadhin00@gmail.com |
| Tania Akter | Rajshahi University | 01745894572@gmail.com |
| Tasmim Hoq | Independent University Bangladesh | tasmimhoque789@gmail.com |
| Nusrat Kamal |  |  |
| Md Nazmul Hassan | Comilla University | nazmulhassan019@gmail.com |
| Md Khaleduzzaman | Patuakhali Science and Technology University | iam.khaleduzzaman@gmail.com |
| Md Riazul Islam Sarker | Jagannath university | remonahmed78@gmail.com |
| Saifur Rahaman | Noakhali Science and Technology University | saifur.nstupharma19@gmail.com |
| Marguba Kamrun | Jahangirnagor University, Savar , Dhaka | margubakamrun28@gmail.com |
| Fariba Chowdhury | Jahangirnagar University | faribachowdhury12@gmail.com |
| Nishat Anjum Eti | Independent university | armstronganjum@gmail.com |
| Nujhat Moonawara | Dhaka University | nujhatmuna@gmail.com |
| Amit Kumar Roy | Beximco Pharmaceuticals Limited | amitkroy1990@gmail.com |
| Sumaiya Farzana Quaderi | Global school and College | sumiju33@gmail.com |
| Sadman Sarar | Patuakhali Science and Technology University | sadmansarar777@gmail.com |
| Md. Naeem Islam | Jahangirnagar University | mnislam344@gmail.com |
| Maria Meha Promi | Rajshahi University | meha.mitsa@gmail.com |
| Md. Saroar Hossen | Sher-e-Bangla Agricultural University | sarwar.bd1995@gmail.com |
| Afsara Jahin Rafi | Jahangirnagar University | rafijahin@ieee.org |
| Arafat Rahman | Jahangirnagar University | a4242r@gmail.com |
| Name | **Institution** | **Email** |
| Nadia Akter | Noakhali Science and Technology University | Nadia.tazin12@gmail.com |
| Israt Jahan Pinky | University of Development Alternative | Israt77uoda@gmail.com |
| Md. Hamidur Rahman | Asian Disaster Preparedness Center (ADPC) | sumon.adpc@gmail.com |
| Asma Akter | National University | [Asmabinteasmu2@gmail.com](mailto:Asmabinteasmu2@gmail.com) |
| Md Nahid Hassan | Jahangirnagar University | mdnahiid6@gmail.com |
| Faria Naznin | Noakhali Science and Technology University | farianaznin586@gmail.com |
| Md. Asiqur Rahman | Sonargaon University | asiqlaw39du@gmail.com |
| Supriya Saha | Jashore University of Science & Technology | Supriyasahajust06@gmail.com |
| Kheirun Nahar Jeni | Noakhali science and technology University | aurorajen98@gmail.com |
| Jahanur Biswas | Jahangirnagar University | jahanur.biswas23@gmail.com |
| Nazibul Islam | Shahjalal University of Science and Technology | shefaul359.islam@gmail.com |
| Md. Ashraful Alam | Noakhali Science and Technology University | alamashraf872@gmail.com |
| Tanzila Azad Mow | Jahangirnagar University | azadmow03@gmail.com |
| Bijoy Krishna Roy Shuvo | Jagannath University, Dhaka | royshuvo168@gmail.com |
| Tanwy Mazumder | Jagannath University | tanwymazumder@gmail.com |
| Mahmuda Sultana Mim | Jahangirnagar University | mahmudamim772@gmail.com |
| Ridoy Ahamed | Drug International Limited | ridoy.ahamed@northsouth.edu |
| Sujeed Debnath | Jahangirnagar University | sujitdn48@gmail.com |
| Md. Kamrul Hasan Kayesh | Bangladesh Agricultural University | akayes9282@gmail.com |
| Syed Musfiqur Rahman | Military Institute of Science & Technology | syedmusfiqur.rahman.17@gmail.com |
| Md Enayatur Rahman | Jahangirnagar University | enayatur.ju@gmail.com |
| Khandaker Bushra Rahman | Noakhali Science and Technology University | tashmi0101@gmail.com |
| Md. Asif Iqbal | Bangladesh University of Professionals | asif92980@gmail.com |
| Md.Naimur Rahman | North South University | emonnsu142@gmail.com |
| Sumon Mitra | IRD Global | sumonmitra1995@gmail.com |
| Julia Akter Meshu | University of Rajshahi | juliaakter.bd@gmail.com |
| Md. Rubel Gazi | Patuakhali science and technology University | gmrubel1995@gmail.com |
| Dr. Md. Moksed Ali | Hamdard University Bangladesh | drmoksedali@gmail.com |
| Md Zahidur Rahman Arnob | Jahangirnagar University | zrarnob@gmail.com |
| Md. Baejid Islam | Jahangirnagar University | nafibaejid@gmail.com |
| Md. Ibrahim Khalil | Noakhali Science and Technology University | imran.nstu007@gmail.com |
| Akibul Islam Chowdhury | Noakhali Science and Technology University | akibul433@gmail.com |
| Sayeda Jahan | Jahangirnagar University | sayeda.jahan09@gmail.com |
| Md Mostafa Kamal | Northeastern Polytechnical University, China | shuvrobbaju@gmail.com |
| Rejina Akter | Jahangirnagar University | rejinadphi@gmail.com |
| Nyeem Ahamed Khan | Chandlee group of industries | nyem22khan@gmail.com |
| Md Mustafizur Rahman | Jahangirnagar University | fizz1336@gmail.com |
| Md Mehedi Hasan | Noakhali science and technology univarsity | rafsanmehedi1001@gmail.com |
| Yeasin Arafath Apu | Noakhali Science And Technology University | yeasinsadad52@gmail.com |
| Md. Suliman | Jagannath University | sulimansiam82@gmail.com |
| Md Symum Korim | Rajshahi University | symumkorim@gmail.com |
| Md.Khaled | Bangladesh University of Business and Technology | imkhaled404@gmail.com |
| Sumaiya Akter | Jahangirnagar University | sumaiyaabeer1319@gmail.com |
| Ankon Das | Noakhali Science and Technology University | ankon2000das@gmail.com |
| Md. Akram Hossain Pavel | Delta Pharma Limited | akrampavel50@gmail.com |
| Kobirul Islam | Dhaka college | 1996kobirulislam@gmail.com |
| Md. Yeasin Arafat | Gov't Unani & Ayurvedic Medical College | arafatimran56@gmail.com |
| Mosa. Nabida Tabassum | RajshahiUniversity | nabidanabu25@gmail.com |
